# Evolving changes in disease biomarkers and risk of early progression in smoldering multiple myeloma

**DOI:** 10.1038/bcj.2016.65

**Published:** 2016-07-29

**Authors:** P Ravi, S Kumar, J T Larsen, W Gonsalves, F Buadi, M Q Lacy, R Go, A Dispenzieri, P Kapoor, J A Lust, D Dingli, Y Lin, S J Russell, N Leung, M A Gertz, R A Kyle, P L Bergsagel, S V Rajkumar

**Affiliations:** 1 Department of Internal Medicine, Mayo Clinic, Rochester, MN, USA; 2 Division of Hematology, Mayo Clinic, Rochester, MN, USA; 3 Division of Hematology and Oncology, Mayo Clinic Arizona, Scottsdale, AZ, USA

## Abstract

We studied 190 patients with smoldering multiple myeloma (SMM) at our institution between 1973 and 2014. Evolving change in monoclonal protein level (eMP) was defined as ⩾10% increase in serum monoclonal protein (M) and/or immunoglobulin (Ig) (M/Ig) within the first 6 months of diagnosis (only if M-protein ⩾3 g/dl) and/or ⩾25% increase in M/Ig within the first 12 months, with a minimum required increase of 0.5 g/dl in M-protein and/or 500 mg/dl in Ig. Evolving change in hemoglobin (eHb) was defined as ⩾0.5 g/dl decrease within 12 months of diagnosis. A total of 134 patients (70.5%) progressed to MM over a median follow-up of 10.4 years. On multivariable analysis adjusting for factors known to predict for progression to MM, bone marrow plasma cells ⩾20% (odds ratio (OR)=3.37 (1.30–8.77), *P*=0.013), eMP (OR=8.20 (3.19–21.05), *P*<0.001) and eHb (OR=5.86 (2.12–16.21), *P*=0.001) were independent predictors of progression within 2 years of SMM diagnosis. A risk model comprising these variables was constructed, with median time to progression of 12.3, 5.1, 2.0 and 1.0 years among patients with 0–3 risk factors respectively. The 2-year progression risk was 81.5% in individuals who demonstrated both eMP and eHb, and 90.5% in those with all three risk factors.

## Introduction

Smoldering multiple myeloma (SMM) is a plasma cell disorder characterized by serum monoclonal protein (M) ⩾3 g/dl and/or 10–60% clonal bone marrow plasma cells (BMPCs), with no evidence of myeloma-defining events or amyloidosis.^1^ It is found in 14% of people with newly diagnosed multiple myeloma (MM),^2^ a disease predicted to account for >2% of all cancer deaths in the United States this year.^3^

Defining the risk of progression from SMM to MM has been heavily studied, with several factors found to predict for high-risk SMM (defined as ⩾50% risk of progression to MM within 2 years).^4^ These include serum M-protein ⩾3 g/dl,^5^ a higher extent of BMPC involvement,^5^ immunoglobulin A (IgA) SMM,^5^ immunoparesis in uninvolved Igs,^6^ increased circulating plasma cells,^7^ cytogenetic abnormalities,^8^ a serum involved/uninvolved free light chain ratio ⩾8^9^ and abnormalities on magnetic resonance imaging^10^ and positron emission tomography–computed tomography.^11^ Identification of such risk factors is important as a randomized trial conducted by the Spanish Myeloma Group showed that early treatment with lenalidomide and dexamethasone delayed progression and improved overall survival in high-risk SMM patients compared with observation, which remains the current standard of care.^12^

There has been some interest in evaluating the impact of changes in disease biomarkers on progression to symptomatic disease. An ‘evolving' pattern in serum M-protein independently predicted for progression to MM in a single-center cohort, with a 2-year progression risk of 45%,^13^ and a similar risk was observed in a SWOG study among patients in whom there was a change in M-protein from <3 to ⩾3 g/dl over a period of 4 months.^14^ However, *post hoc* analysis from the observation arm of the Spanish trial suggested that progression risk was comparable between patients with and without evolving changes in M-protein.^15^

Based on these considerations, we sought to evaluate the impact of evolving changes in SMM disease biomarkers on risk of progression to MM. We specifically aimed to try and use such changes to identify a cohort of ultra-high-risk SMM with ⩾80% risk of progression to MM within 2 years (that is, early progression), a threshold that has been deemed by the International Myeloma Working Group (IMWG) to meet diagnostic criteria for MM.^1^

## Materials and methods

### Study cohort

We identified all patients diagnosed with SMM at our institution between 1973 and 2014 (*n*=1253) from the Mayo Clinic institutional review board-approved electronic database. Only those meeting the revised 2014 IMWG diagnostic criteria for SMM^1^ were included, and those who received myeloma-specific therapy as part of a therapeutic strategy to delay progression to MM were excluded. Patients who did not have adequate follow-up (that is, a minimum of two data points before confirmed progression to MM) to permit study of evolving changes in at least one SMM-specific biomarker (see below) were also excluded.

### Biomarkers studied, definitions of evolving changes and outcomes

For each patient, baseline levels of various SMM parameters (serum M-protein, percentage clonal BMPCs, Igs, involved/uninvolved free light chain ratio, creatinine, calcium, hemoglobin (Hb), lactate dehydrogenase and β2-microglobulin) at diagnosis were abstracted from the electronic medical record. Levels of SMM-specific biomarkers (serum M-protein, involved Ig, Hb, calcium and creatinine) at each follow-up for the first 3 years after diagnosis were also abstracted to permit study of evolving changes. Immunoparesis was defined as reduction below the lower limit of normal in two uninvolved Igs.

Evolving change in monoclonal protein level (eMP) was defined as ⩾10% increase in serum M-protein and/or Ig within the first 6 months of diagnosis (only if M-protein was ⩾3 g/dl) and/or ⩾25% increase in serum M-protein and/or Ig within the first 12 months of diagnosis (for any level of M-protein), with a required minimum increase of either 0.5 g/dl in M-protein or 500 mg/dl in Ig (or both). We used changes in Ig conjunctively with M-protein to study evolving change in monoclonal disease burden given that both of them represent outputs from a clonal plasma cell population. Evolving change in hemoglobin level (eHb) was defined as ⩾0.5 g/dl decrease within the first 12 months of diagnosis. We also studied changes in calcium and creatinine, but were unable to find consistent patterns to produce a candidate definition of an evolving change in these two variables.

Time to progression (TTP) was defined as duration between confirmed diagnosis of SMM (as per revised 2014 IMWG criteria) and diagnosis of MM or commencement of myeloma-directed therapy for symptomatic disease by the treating physician, or censored at last follow-up. Overall survival was defined as duration between SMM diagnosis and death, or censored at the last follow-up date.

### Statistical analysis

Univariate logistic regression analyses were used to test the association between variables of interest and the risk of early progression to MM. Variables achieving a significance level of *P*<0.2 on univariable analysis were included in the multivariable logistic regression analysis. Survival and TTP analyses were performed using the Kaplan–Meier method, and the log-rank test was used to make comparisons between groups. All tests were two sided with *P-*values of <0.05 considered to be significant. Statistical analysis was performed using SPSS v.20 (IBM Corp., Armonk, NY, USA).

## Results

A total of 190 patients were included in the analysis (Table 1). Median age at SMM diagnosis was 64 years, and the majority of patients (55%) were male. Of the cohort, 66.3% had a serum M-protein of <3 g/dl at diagnosis and BMPC was ⩾10% in the vast majority (95.3%) of patients. Of the patients, 75.8% had IgG SMM, with most of the remainder having IgA disease; immunoparesis was observed in 61.6% of patients.

A total of 134 individuals (70.5%) progressed to MM over a median follow-up of 10.4 years, with a median TTP of 3.9 years (95% confidence interval 2.8–5.1). Of those who progressed, 61 patients (45.5%) did so within 2 years of diagnosis, and progression events were primarily due to anemia (44.0%) or bony lesions (36.6%). Median overall survival in the entire cohort was 10.0 years (8.9–11.1).

### Evolving changes in monoclonal protein and hemoglobin

Table 2 provides the definition used for eMP and eHb and Table 3 shows the numbers of patients who met these criteria. A total of 58 patients (30.5%) demonstrated eMP—of these, 24 (41.4%) met criteria for evolving change in M-protein, 15 (25.9%) for evolving change in Ig and 19 (32.8%) meeting criteria for evolving changes in both M-protein and Ig. In the vast majority of patients (84.5%), confirmation of the change or progression to MM was seen at the next follow-up. In addition, 48 patients (25.3%) displayed eHb, and the change was either confirmed, or progression to MM determined, at next follow-up in all of these individuals.

### Predictors of early (⩽2 years) progression to MM

Table 4 shows the results of uni- and multivariable logistic regression analyses assessing predictors of progression to MM within 2 years of SMM diagnosis. On univariable analysis, male sex (odds ratio (OR)=2.39 (1.26–4.54), *P*=0.008), BMPC ⩾20% (OR=4.41 (2.26–8.57), *P*<0.001), eMP (OR=9.55 (4.62–19.73), *P*<0.001) and eHb (OR=8.25 (3.92–17.36), *P*<0.001) predicted for early progression. All four variables remained independent predictors of early progression on multivariable analysis (male sex: OR=3.51 (1.32–9.29), *P*=0.012; BMPC ⩾20%: OR=3.37 (1.30–8.77), *P*=0.013; eMP: OR=8.20 (3.19–21.05), *P*<0.001; eHb: OR=5.86 (2.12–16.21), *P*=0.001).

### Risk model for early progression, and 2-year progression risks

**Figure 1:**
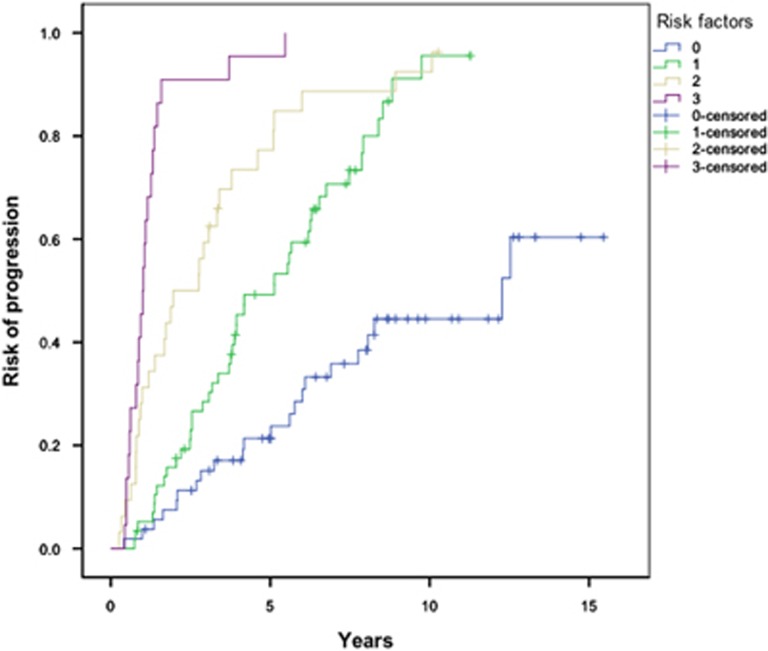
Risk of progression in SMM patients, stratified by the number of risk factors (eMP, eHb and BMPC ⩾20%) at diagnosis. *P*<0.001.

Based upon the three disease-specific variables independently predicting for early progression (BMPC ⩾20%, eMP and eHb), a risk model for progression was constructed based upon the number of risk factors (0–3) for each patient at diagnosis (Figure 1). Median TTP in patients with none (*n*=54), one (*n*=58), two (*n*=32) and three (*n*=22) risk factors was 12.3 (7.1–17.5), 5.1 (3.3–6.9), 2.0 (0.5–3.4) and 1.0 years (0.8–1.2) respectively (*P*<0.001).

Table 5 shows the rates of early progression among patients with different disease-specific risk factors. The 2-year progression risks were 63.8% and 64.6% in patients displaying eMP and eHb respectively; among individuals who displayed both eMP and eHb, the early progression risk was 81.5%, and this increased to 90.5% in patients with all 3 risk factors (BMPC ⩾20%, eMP and eHb).

## Discussion

The natural history of SMM has been well defined, with a risk of progression to MM of 10% per year for the first 5 years, 3% per year for the next 3 years and 1% per year thereafter.^5^ Several factors predicting for a higher risk of progression have been identified, leading to the development of risk models,^6, 9^ although there remains discordance in their predictive accuracy.^16^ The need to improve the risk stratification of SMM patients has arisen as a result of the development of more effective and less toxic treatments in myeloma that may allow SMM patients the opportunity to be treated before the herald of end-organ damage. Indeed, randomized data have shown a progression and survival benefit in high-risk SMM patients treated with lenalidomide and dexamethasone compared with observation,^12^ and additional trials utilizing novel agents are currently underway in high-risk patients.

In 2014, the IMWG updated the diagnostic criteria for MM by removing the requirement for end-organ damage to define malignancy, and called for the development of additional biomarkers associated with a risk of progression of SMM to MM of at least 80% within 2 years, to enable their addition to diagnostic criteria for MM.^1^ In this study, we have shown that SMM patients with evolving changes in their monoclonal protein burden (defined by changes in M-protein and/or involved Ig) and Hb are at >80% risk of progressing to MM within the first 2 years of diagnosis, and that patients with both evolving changes and a BMPC of ⩾20% have a 2-year progression risk of >90%. Although these findings require validation, these data provide the first step toward incorporating evolving changes in commonly measured disease biomarkers in diagnostic definitions for myeloma or as an indication for treatment in SMM.

The first data on how changes in M-protein impact on progression came from the recognition of an evolving subtype among SMM patients treated at a single institution in Spain.^17^ Rosinol *et al.*^17^ found that TTP to MM was significantly higher among patients in whom serum M-protein increased in each of the first two consecutive follow-up visits after SMM diagnosis compared with patients who did not experience such a change; moreover, this evolving subtype was the only factor that independently predicted for shorter TTP. Subsequent work from the same institution showed that an evolving change in M-protein (defined as ⩾10% increase in the first 6 months if M-protein was ⩾3 g/dl, or a progressive increase in each of the annual consecutive measurements during a period of 3 years if M-protein was <3 g/dl) was an independent predictor of progression to MM, with an associated 2-year progression risk of 45%.^13^ Aside from these studies, the only other data on evolving changes in SMM come from a subgroup analysis within the SWOG S0120 prospective study on asymptomatic monoclonal gammopathies, with a 33.3% 2-year progression risk seen among the 6 patients in whom M-protein increased from <3 to ⩾3 g/dl in a 4-month period.^14^

In this regard, our study represents a valuable contribution to the literature by confirming that evolving changes in monoclonality (measured by M-spike and/or involved Ig) are an independent risk factor for progression to MM and, to our knowledge, being the first to show that evolving change in Hb is also predictive for early progression. In addition, our definitions of evolving change allow for both sensitivity and robustness in identifying patients with this phenotype, given that they can be measured within the first year of SMM diagnosis, can be achieved through either changes in M-protein or involved Igs (when the M-protein is too small) and have a minimum threshold to ensure that clinically insignificant findings (for example, from small rises meeting percentage thresholds in M-protein or Ig) are ignored.

The processes underlying progression of SMM to MM provide the biologic explanation that underpins our findings. Generation of a plasma cell clone arises from hyperdiploidy and translocation of the Ig heavy-chain locus that leads to cyclin D dysregulation and initiation of clonal plasma cell evolution.^18^ Further genetic, epigenetic and karyotypic abnormalities drive expansion of the plasma cell clone, allowing transformation from the precursor states of monoclonal gammopathy of undetermined significance and SMM toward symptomatic MM.^19^ It is therefore apparent that these events are associated with increasing production of M-protein and the involved Ig, with the expansion of clonal plasma cells leading to bone marrow suppression and giving rise to a progressive reduction in Hb. Consequently, identifying changes in these parameters provides the ability to identify patients with more aggressive disease biology, thereby allowing these individuals to avail from therapy before they suffer end-organ damage.

The main strength of our study lies in the relatively large number of patients included, representing the largest cohort on evolving changes in SMM that has been published to date, as well as the long follow-up available. In addition, we used the latest diagnostic criteria for SMM to ensure that patients with parameters such as BMPC ⩾60% and free light chain ⩾100, which have recently been categorized as being diagnostic for MM,^1^ were excluded. Furthermore, our definition of evolving change are simple and permit easy calculation for clinicians, as well as being in keeping with similar criteria for disease progression in MM^20^ and previous definitions of evolving changes.^13^

Nevertheless, there are certain limitations of this study in addition to those inherent to a single-center, observational, retrospective study. First, >70% of our cohort progressed to MM during the study period, with >30% progressing within 2 years of diagnosis. Given that high-risk SMM is defined as ⩾50% risk of progression within 2 years,^4^ this suggests our cohort was particularly high risk at baseline and may therefore limit the generalizability of our findings. Second, it may be argued that a reduction in Hb of ⩾0.5 g/dl, which constituted an evolving change, is too small to reflect a significant change in disease status, as well as potentially falling within the margin of error provided by laboratory variability and changes in hydration status. However, our intention was to balance sensitivity and specificity in producing a cutoff for evolving change; moreover, the validity of utilizing the ⩾0.5 g/dl threshold was shown by the change either being confirmed on next measurement or progression to MM being demonstrated at next follow-up in all 48 patients who met this criterion.

In summary, this study identified that evolving changes in Hb and M-protein and/or involved Ig, in addition to BMPC ⩾20%, were independent predictors of early progression in a large single-center cohort of patients with SMM. Individuals displaying evolving changes in both monoclonality and Hb, with or without a clonal BMPC of ⩾20%, had >80% risk of progression to MM within 2 years of diagnosis, meeting the IMWG threshold for a diagnosis of MM. We hope that following independent validation, our findings can be used to update the diagnostic criteria for MM, or to be considered as an indication for therapy of SMM.

## Figures and Tables

**Table 1:** Baseline characteristics of 190 patients at SMM diagnosis

	*N*
Age, median (range)	64 (30–83)
	
*Sex (%)*	
Male	104 (54.7)
Female	86 (45.3)
	
*Serum M-protein (%)*	
<3 g/dl	126 (66.3)
⩾3g/dl	52 (27.4)
Not available	12 (6.3)
	
*Clonal bone marrow plasma cells (%)*	
<10	7 (3.7)
10–19	90 (47.4)
20–59	91 (47.9)
Not available	2 (1.1)
	
*Immunoglobulin subtype (%)*	
IgG	144 (75.8)
IgA	35 (18.4)
IgM	2 (1.1)
IgD	2 (1.1)
Biclonal	1 (0.5)
Unknown	6 (3.2)
	
*Immunoparesis (%)*	
Present	117 (61.6)
Absent	33 (17.4)
Not available	40 (21.1)
Hemoglobin (g/dl), median (range)	12.7 (7.2–17.1)
	
*Involved/uninvolved FLC ratio (%)*	
<8	45 (23.7)
⩾8	42 (22.1)
Not available	103 (54.2)
LDH, median (range), units/l	143 (3–451)
β2-microglobulin, median (range), μg/ml	2.4 (1–17.6)
Progression to multiple myeloma (%)	134 (70.5)
Median, years (95% CI)	3.9 (2.8–5.1)
⩽2 Years	61 (45.5)
>2 Years	73 (54.5)
	
*Progression events (%)*	
Anemia	59 (44.0)
Bone lesions	49 (36.6)
Renal failure	16 (11.9)
Other	10 (7.5)

Abbreviations: CI, confidence interval; FLC, free light chain; Ig, immunoglobulin; LDH, lactate dehydrogenase; SMM, smoldering multiple myeloma.

**Table 2:** Definitions of evolving changes in monoclonal protein (eMP) and hemoglobin (eHb)

*Criteria*	*Definition*
Evolving monoclonal protein level (eMP)	⩾10% Increase in serum M-protein and/or involved immunoglobulin level within the first 6 months of diagnosis (only if baseline M-protein was ⩾3 g/dl) *and/or* ⩾25% increase in serum M-protein and/or involved immunoglobulin level within the first 12 months of diagnosis (for any level of M-protein) *plus minimum increase of* 0.5 g/dl in M-protein or 500 mg/dl in immunoglobulin or both
Evolving hemoglobin level (eHb)	⩾0.5 g/dl Decrease in hemoglobin within the first 12 months of diagnosis

**Table 3:** Evolving changes in monoclonal protein (eMP) and hemoglobin (eHb)

*Parameter*	*N*
*eMP (%)*	
Present	58 (30.5)
Evolving M-protein	24 (41.4)
Evolving Ig	15 (25.9)
Evolving M-protein and Ig	19 (32.8)
Confirmed on next measurement^a^	49 (84.5)
Absent	122 (64.2)
Not evaluable	10 (5.3)
	
*eHb (%)*	
Present	48 (25.3)
Confirmed on next measurement^a^	48 (100)
Absent	127 (66.8)
Not evaluable	15 (7.9)

Abbreviation: Ig, involved immunoglobulin.

a Including patients who progressed at next follow-up date.

**Table 4:** Univariable and multivariable logistic regression analyses of predictors of progression to MM within 2 years of SMM diagnosis

	*Univariable*		*Multivariable (*n*=156)*	
	*OR (95% CI)*	P-*value*	*OR (95% CI)*	P-*value*
Age	1.01 (0.98–1.04)	0.626	—	—
Male sex	2.39 (1.26–4.54)	0.008	3.51 (1.32–9.29)	**0.012**
BMPC ⩾20%	4.41 (2.26–8.57)	<0.001	3.37 (1.30–8.77)	**0.013**
M-protein ⩾3 g/dl	1.68 (0.86–3.30)	0.129	0.41 (0.13–1.31)	0.132
IgA SMM	1.69 (0.80–3.58)	0.171	1.09 (0.36–3.32)	0.883
Immunoparesis	1.59 (0.63–3.99)	0.328	—	—
FLC ⩾8	1.57 (0.60–4.10)	0.358	—	—
eMP	9.55 (4.62–19.73)	<0.001	8.20 (3.19–21.05)	**<0.001**
eHb	8.25 (3.92–17.36)	<0.001	5.86 (2.12–16.21)	**0.001**
LDH>ULN	0.30 (0.04–2.49)	0.266	—	—
β2-microglobulin >3.5 μg/ml	0.86 (0.34–2.22)	0.760	—	—

Abbreviations: BMPC, clonal bone marrow plasma cell; 95% CI, 95% confidence interval; OR, odds ratio; eHb, evolving hemoglobin; eMP, evolving monoclonal protein; FLC, free light chain; IgA, involved immunoglobulin A; LDH, lactate dehydrogenase; MM, multiple myeloma; SMM, smoldering multiple myeloma; ULN, upper limit of normal. Bold values indicate statistically significant on multivariate analysis.

**Table 5:** Risk of early progression (⩽2 years) in SMM patients with disease-specific risk factors

*Risk factor*	*Proportion of patients progressing within 2 years (%)*
BMPC ⩾20%	44/91 (48.4)
eMP only	37/58 (63.8)
eHb only	31/48 (64.6)
Both eMP and eHb	22/27 (81.5)
eMP, eHb and BMPC ⩾20%	19/21 (90.5)

Abbreviations: BMPC, clonal bone marrow plasma cells; eHb, evolving hemoglobin; eMP, evolving monoclonal protein; SMM, smoldering multiple myeloma.
